# Silver Bionanocomposites as Active Food Packaging: Recent Advances & Future Trends Tackling the Food Waste Crisis

**DOI:** 10.3390/polym15214243

**Published:** 2023-10-27

**Authors:** Federico Trotta, Sidonio Da Silva, Alessio Massironi, Seyedeh Fatemeh Mirpoor, Stella Lignou, Sameer Khalil Ghawi, Dimitris Charalampopoulos

**Affiliations:** 1 Metalchemy Limited., 71-75 Shelton Street, London WC2H 9JQ, UK; sids@metalchemy.tech (S.D.S.); am@metalchemy.tech (A.M.); 2 Department of Food and Nutritional Sciences, University of Reading, P.O. Box 226, Whiteknights, Reading RG6 6AP, UKs.lignou@reading.ac.uk (S.L.); s.khalilghawi@reading.ac.uk (S.K.G.); d.charalampopoulos@reading.ac.uk (D.C.)

**Keywords:** bionanocomposites, food packaging, silver nanoparticles, bioplastics, colloidal silver, green chemistry, biopolymers, food waste, AgNPs

## Abstract

Food waste is a pressing global challenge leading to over $1 trillion lost annually and contributing up to 10% of global greenhouse gas emissions. Extensive study has been directed toward the use of active biodegradable packaging materials to improve food quality, minimize plastic use, and encourage sustainable packaging technology development. However, this has been achieved with limited success, which can mainly be attributed to poor material properties and high production costs. In the recent literature, the integration of silver nanoparticles (AgNPs) has shown to improve the properties of biopolymer, prompting the development of bionanocomposites. Furthermore, the antibacterial properties of AgNPs against foodborne pathogens leads towards food shelf-life improvement and provides a route towards reducing food waste. However, few reviews have analyzed AgNPs holistically throughout a portfolio of biopolymers from an industrial perspective. Hence, this review critically analyses the antibacterial, barrier, mechanical, thermal, and water resistance properties of AgNP-based bionanocomposites. These advanced materials are also discussed in terms of food packaging applications and assessed in terms of their performance in enhancing food shelf-life. Finally, the current barriers towards the commercialization of AgNP bionanocomposites are critically discussed to provide an industrial action plan towards the development of sustainable packaging materials to reduce food waste.

## 1. Introduction

**Figure 1:**
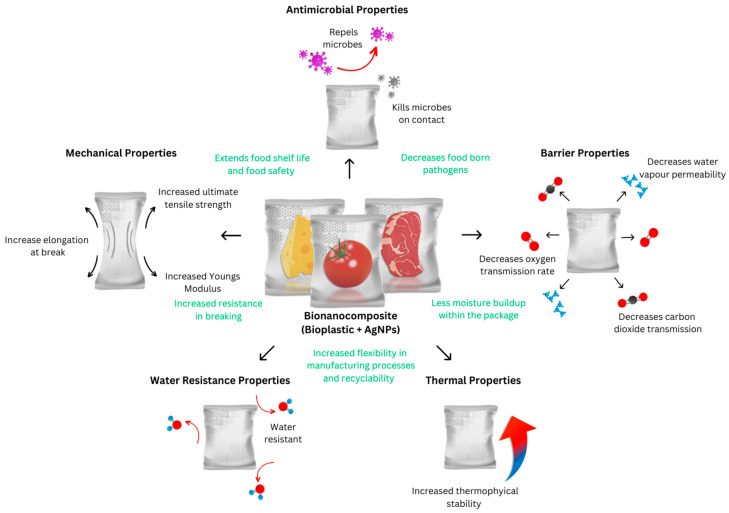
Visual abstract outlining the enhanced properties of bionanocomposites. Green text summarizes the key benefits of the bionanocomposites for food packaging applications.

Food packaging is a critical component of food technology that deals with the protection and preservation of diverse food products [1]. It has been reported that food packaging represented a global market size of £303 Billion in 2021 with a compound annual growth rate (CAGR) of 5.5% until 2030, and formed about 69% of the overall consumer packaging market [2]. Petrochemical plastics have achieved widespread success as packaging materials in the sector, owning 99% of the market share due to optimal properties such as oxygen barrier capabilities, high tensile, and tear strength. Other characteristics, such as a high Water Vapor Transmission Rate and biodegradability, is less prevalent in packaging, with biodegradability found in only 0.64% of all plastic materials. All these properties guard against external degradation agents and prevent the internal loss of nutrition in food products, assuring food quality at every level of the supply chain, from producers to end users (Figure 1).

**Figure 2:**
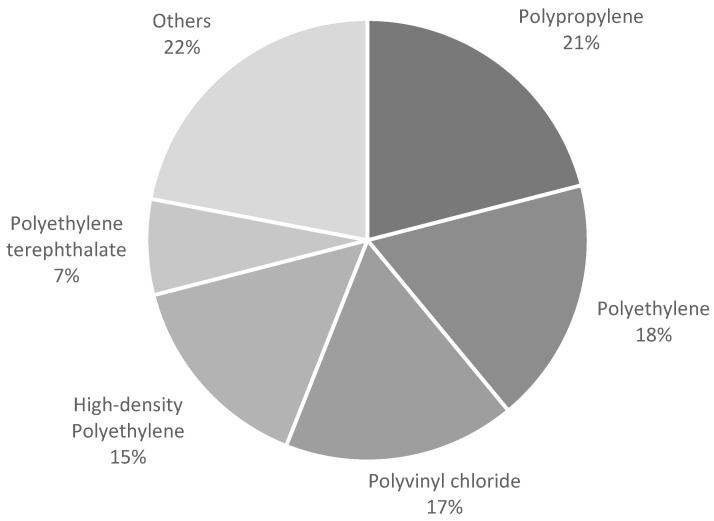
Global market distribution of synthetic plastics [1].

As shown in Figure 2, synthetic plastic polymers such as polypropylene (21%), polyethylene (18%), polyvinyl chloride (17%), high-density polyethylene (15%), and polyethylene terephthalate dominate the food packaging market globally [1]. Between 1950 and 2015, an estimated 7.8 billion tons of plastics were created worldwide, with approximately 4.6 billion tons ending up in landfill or being wasted [3]. Polyethylene, the most manufactured and discarded synthetic polymer globally, is the major generator of two greenhouse gases—methane and ethylene [4]. Methane emissions contribute to climate change and can harm aquatic life by changing the oxygen levels and pH of water, whereas ethylene emissions can be hazardous to plants and animals and have an impact on crops and biodiversity, and there is some evidence that it may play a role in cancer development [4]. 

Plastics account for 10% of the global oil output, with single-use plastics accounting for more than one third of all plastics produced in 2017 [5]. Several environmentally hazardous disposal methods, such as incineration and landfill, are currently being employed to deal with the overflow of plastics [4]. As a result, sustainable, safe, and non-toxic food packaging options are highly desirable to ensure a transition to more environmentally friendly packaging materials in the food industry.

Bioplastics represent an innovative category of plastics derived from natural sources, such as chitosan, agar, alginate, and polylactic acid (PLA), among others. They are positioned as an environmentally friendly alternative to non-biodegradable synthetic plastics due to their reduced reliance on fossil fuels, faster biodegradability, and lower carbon footprint [6]. Bioplastics are made of biopolymers and biodegradable reinforcing agents [7]. The ability of bioplastics to return to the ecosystem, either through the natural breakdown of organic waste by microorganisms or composting, rather than accumulating in landfills, is an important differentiating factor compared to non-biodegradable synthetic plastics. 

Even though bioplastics provide an alternative to synthetic packaging, they are regularly combined with petrochemical plastics. This is due to their weak mechanical qualities and moisture sensitivity, which are listed as contributing causes to their restricted utilization in food packaging [6]. To overcome the challenges around bioplastics, extensive research has been conducted to embed nanomaterials in food packaging materials, leading to the development of active packaging materials. Several metallic nanoparticles, most notably silver, aluminum, and zinc, have been shown to improve qualities such as tensile strength, Water Vapor Permeability, and biocidal activity [8]. Moreover, silver nanoparticles (AgNPs) have been found to have a strong antibacterial effect against foodborne pathogens such as bacteria, parasites, and viruses [9]. 

The antimicrobial activity of AgNPs in food packaging can help tackle two major global challenges:

- Food and beverage waste: Excess food production used to compensate for waste could be used to help feed the 811 million people worldwide experiencing chronic undernourishment [10];
- Foodborne infections: 550 million cases and 230,000 deaths worldwide each year could be avoided by providing sustainable and effective food packaging technology [9].

These challenges are aligned with the United Nations Sustainable Development Goal 12 to reduce food waste along production and supply chains in order to promote a more sustainable economic model [11]. Food waste is typically generated by food products which have a short shelf-life; studies have shown a wide spectrum of microorganisms being responsible for food deterioration [9], increasing the challenge to finding a one-stop solution to prevent highly nutritious foods from degrading as quickly, especially in warmer climates. As a result, $1.2 trillion is wasted globally each year from food and beverage detorioration [10]. It is believed that over 1.3 billion tons, or one third of all food produced for human use, is wasted annually [10]. This accounts for up to 10% of global greenhouse gas emissions [10].

While the main focus of current research has been to investigate the incorporation of AgNPs in bioplastics to improve their physical properties, only a limited amount of studies have looked into how these improvements affect food packaging in a holistic manner.

Due to the excellent performance of silver bionanocomposites in antibacterial activity and their ability to improve the physico-chemical properties of bioplastics, the purpose of this literature review is to critically examine key technological advances that are relevant to food packaging. These properties include the antimicrobial activity, barrier properties (Water Vapor Transmission Rate (WVTR, WVP), and Oxygen Transmission Rate (OTR)), mechanical properties (Ultimate Tensile Strength (UTS) and Elongation at Break (EaB)), thermal properties, and water resistance (WS, CA) of bionanocomposites containing AgNPs. Specifically, we will focus on the combination of AgNPs with agar and/or PLA and their applications as active materials in food packaging, since they represent the most exploited and promising materials for the development of advanced bioplastics. We also explore the latest developments in the antibacterial activities of silver bionanocomposites. In the last sections of this critical review, we discuss the challenges and environmental impact of silver bionanocomposites, aligning our discussion with current and evolving regulatory frameworks. The nature of this fast-paced and advanced field often leads to fragmented and conflicting literature; thus, this literature review serves to consolidate and clarify the current state of knowledge, contributing to the further understanding of silver bionanocomposites. Additionally, we critically analyze three barriers to the commercialization of these materials including a scalability, regulatory, and environmental analysis outlining the key obstacles that need to be overcome for these advanced materials to become widely available in the industry.

## 2. Silver Nanoparticles as an Active Additive

A common approach to enhancing food safety is to embed an active ingredient within a packaging material which not only inhibits microbial growth, but also enhances Water Vapor Permeability to extend the shelf-life [9]. This is achievable through nanomaterials such as AgNPs, which have a higher surface area-to-volume ratio with respect to their bulk counterpart, allowing them to easily interact with and bond to other materials. 

**Figure 3:**
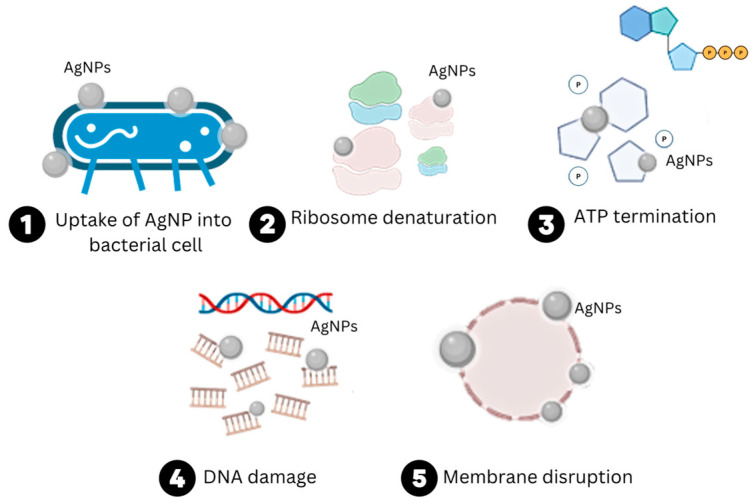
Various antimicrobial activity mechanisms of AgNPs. (1) Entry of the AgNPs into the cell membrane of the bacterial cells; (2) ribosome denaturation; (3) ATP termination; (4,5) membrane disruption; DNA damage; (5) rupture of the cell membrane [9].

Hence, when embedding silver nanoparticles in biopolymers, they interact with:

- Gases, such as oxygen and carbon dioxide, which increase barrier capabilities [12];
- The polymer matrix of the film, forming a network of strong bonds that improve mechanical and barrier properties through weak and covalent interaction, assuring their adhesion within the biopolymers [13];
- Bacteria and other microorganisms, which inhibit their growth [9]. The mechanism of action of AgNPs against bacteria is illustrated in Figure 3;
- UV radiation, reducing the UV penetration through the biopolymer by means of their strong scattering behaviour [14].

It is important to note that the intake of AgNPs into mammalian cells is size dependent, with the aggregation of smaller-sized nanoparticles (<10 nm) causing cytotoxicity to cells [15]. Therefore, packaging manufacturers can counteract the effects of AgNPs in human cells by tuning the size of AgNPs as well as embedding them into bioplastic polymer matrices to form bionanocomposite materials.

The antimicrobial activity of AgNPs, as presented in Figure 3, is influenced by key parameters, including the shape, size, and surface charge of the nanoparticle. Enhanced antimicrobial activity has been demonstrated in spherical and triangular-shaped AgNPs in comparison to cubic, platelet, decahedron, and other shapes, as increasing the surface area increases NPs’ reactivity with the microorganism’s cell membrane [16,17]. Moreover, previous research has demonstrated high antimicrobial properties for AgNPs sized between 1 and 30 nm. Additionally, the AgNPs’ surface charge, conferred by their coating agent, influences their interaction with biological molecules. This includes their uptake by microorganism cells, a crucial aspect governing their antimicrobial mechanism [16].

**Figure 4:**
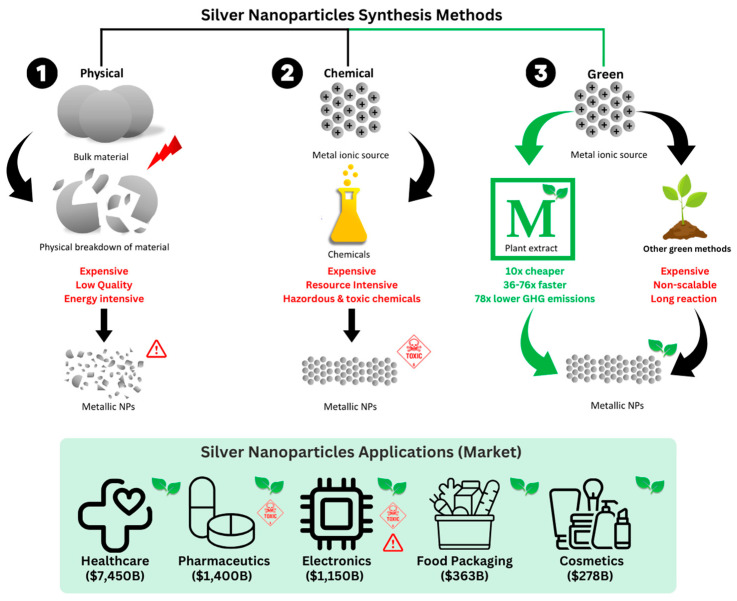
Methods for AgNPs synthesis. (1) Physical: In this method, the bulk material, such as silver foil, is broken down utilizing high energy and material resources; however, the produced AgNPs lack uniform size and shape, the most used methods comprise evaporation-condensation and laser ablation; (2) Chemical: This process commonly involves the metal ionic sources being reduced by a reducing agent and stabilized by a capping agent to produce AgNPs of defined size and shape. However, these use toxic chemicals such as sodium borohydride; (3) Biological: These methods either use microbes or plant extracts to carry out bio-reduction of ionic solutions as well as use biological molecules to stabilize the final AgNPs to produce defined shape/size NPs, without the use of hazardous chemicals [18].

The synthesis method of AgNPs will influence their final physical properties, therefore impacting their antimicrobial efficacy. AgNP production methods can be divided into three synthesis routes: physical, chemical, and biological, as summarized in Figure 4 [18]. To achieve control over AgNPs’ size and morphology, a number of chemical and biological approaches constitute viable options. However, compared to chemical methodologies, biological methods are rapidly becoming the preferred synthesis route due to the absence of hazardous chemicals in their production [18]. Furthermore, biological methods do not require the addition of stabilizing agents during AgNPs’ synthesis, since many of the commonly investigated natural compounds can act both as reducing and capping agents. This enables the development of simpler and more cost-effective reaction strategies compared to chemical methods.

Hence, the selection of the AgNPs’ synthesis method, their characteristics, and their concentration within the bionanocomposite are critical for understanding how to extend food shelf-life, as AgNPs exert different antimicrobial activities and physical properties depending on the bionanocomposite formulation.

## 3. Formulation and Bionanocomposites Manufacturing

Bionanocomposites are composite materials comprising two fundamental components: biopolymers that constitute the bioplastic matrix and embedded nanostructures capable of imparting unique properties which re-enforce the polymeric material. These nanostructures can be organic, encompassing polysaccharides, proteins, or synthetic colloids, or inorganic, including substances such as silica, noble metal oxides, or ceramics. Various bioplastic formulations have been produced in the literature. In particular, polysaccharide-based bionanocomposites are the most investigated due to their unique chemical–physical properties, and relative low cost provided by the abundance of their main sources. Indeed, polysaccharides are naturally abundant, are generally non-toxic and circular, and meet the criteria for packaging production [19]. These biopolymers, unlike lipids which are commonly subjected to peroxidation reactions leading to the loss of their main structure, exhibit a higher thermal stability. However, they are highly susceptible to moisture and have limited mechanical resilience [19]. These include agar [20], chitosan [13], and hydroxypropyl methylcellulose (HPMC) [21] in combination with multiple components such as gum tragacanth/HMPC/beeswaxes (GT/HMPC/BW) [22] and agar/banana powder [23]. Understanding their formulation is critical in order to develop optimal bionanocomposite materials, as their formulation will impact the final material properties as well as our understanding of how AgNPs interact with the polymers themselves.

Physical properties that affect food safety and shelf life must be considered when designing food packaging.

To address these issues, three solutions have been suggested:

1. The addition of different reinforcing chemicals into polysaccharide matrices such as cellulose, lignocellulose, or micro/nanocrystals [24];
2. The combination of different polymers which produce blends or multilayer films, as presented in Table A1 [25];
3. The inclusion of inorganic additives, such as AgNPs [19,25].

Furthermore, plasticizers are commonly used in bioplastics to overcome brittleness and prevent cracking and chipping of the biopolymer during handling and storage. Plasticizers are substances with a low molecular weight and high volatility, such as glycerol or sorbitol. These can reduce intermolecular interactions and boost polymeric chain mobility, resulting in a drop in the material glass transition temperature via protein structural change [26]. It has been demonstrated that adding oleic acid to glycerol improves the mechanical and barrier properties of edible films and coatings [27]. This mixture creates polyglycerol-esters through the esterification of pendant hydroxyl groups with fatty acids. Polyglycerol-esters are commonly employed as additives, modifiers, and emulsifiers in products that contain immiscible food ingredients [28]. The main bioplastic formulations are presented in the table presented in Table A1.

The selection of components is critical to the food packaging quality of the bionanocomposite, where the base formulation of the bioplastic will not only define the starting point of the physical, chemical, and biological properties of the film, but is also critical in understanding the interaction of the AgNPs with the bioplastic formulation itself, which also depend on how the AgNPs are integrated into the biopolymer matrix. 

AgNPs can be embedded into bionanocomposites using a variety of techniques, including in situ synthesis [29], solution blending (melt blending and solution casting) [30], and electrospinning [31]. The first method involves the synthesis of AgNPs within the biopolymer itself by adding a silver salt to a solution containing a reducing agent before its addition into a bioplastic material. Melt blending mixes AgNPs with a bioplastic in its molten state. On the other hand, in the solution casting method, a bioplastic is dissolved in a solvent, allowing the nanoparticles to be dispersed in the solution and mixed with the polymer before being cast into sheet materials. Finally, AgNPs can be electrospun to produce nanofibers and then integrated into a biopolymer solution. The benefits and drawbacks of each method are summarized in Table 1.

Overall, the choice of embedding technique will depend on the specific packaging material applications and the desired properties of the final product as well as the economic viability of such a process. For example, thermal extrusion methods will be the preferred choice for thermoplastic polymers such as Poly (Lactic Acid) (PLA) bionanocomposite production but not for agar-cellulose materials for which in-situ synthesis and mixing are preferred due to the high water affinity of AgNPs once stabilised by hydrophilic capping agents. 

A fundamental aspect to assure an increase in bionanocomposites activities is represented by the AgNPs’ uniform distribution within the biocomposites, in order to maintain an homogeneous action over the bionanocomposites’ surface and avoid AgNP aggregations, which can cause a lack of activities. To achieve homogeneity and uniform dispersion, manufacturers employ different techniques during the processing of the material:

- The use of dispersant or surfactant [33];
- Mechanical mixing during the extrusion process for thermoplastic polymers [34];
- Sonication to break down agglomerates and ensure proper dispersion [35];
- The fine tuning of processing conditions, such as temperature, pressure, and mixing time to optimize AgNP dispersion.

Finally, the AgNPs’ high stability at different conditions, in particular with regard to pH and temperature, is a required feature when they are embedded within bionanocomposites used in food packaging materials to ensure food safety, regulatory compliance, consumer acceptance, and the long-term performance of the packaging. AgNP aggregates can lead to changes in material performance, compromising the integrity and functionality of the packaging [36]. 

## 4. Characterization of Bionanocomposites 

### 4.1. Antimicrobial Activity of Ag Bionanocomposite Materials

AgNPs have been proven to be broadly efficient against common food bacteria such as *Escherichia coli*, *Staphylococcus aureus*, and *Pseusomonas aeruginosa*. The average minimum inhibitory concentration (MIC) of AgNPs for *E. coli* was found to be 10.85 μg/mL [30]. Similarly, AgNPs’ MIC of S. aureus was found to be 14.39 μg/mL [30]. Finally, the average AgNP MIC of *P. aerugionsa* was reported to be 6.41 μg/mL [37]. This body of evidence implies that S. aureus is the most vulnerable of these widespread bacterial species to AgNPs, and have the potential to cause serious food poisoning, especially in milk and cheeses [9]. AgNPs have also shown antimicrobial efficacy against a variety of fungi and viruses often found in food [38], including *Cladosporium*, *Aspergillus*, and *Norovirus*, which are prevalent in carbohydrate-based foods, chilled meat, leafy greens, and shellfish, respectively.

A thorough review of bionanocomposite using AgNPs and their antimicrobial activity is presented in Table 2. AgNPs have a strong bactericidal rate on common food pathogens such as *S. aureus*, *E. coli*, *Listeria monocytogenes*, and *Salmonella typhimurium*. To further optimize the use of AgNPs, the stronger the antibacterial effect of the AgNPs, the lower the concentration of AgNPs that is required to achieve the same result [30].

The literature has generally outlined that smaller particle sizes increase the surface area-to-volume ratio, therefore having a higher antimicrobial activity [16]. This is shown by comparing the results of Argudín et al. [39] and Elgorban et al. [40], for which the same AgNP concentration of 1% (wt/wt) was integrated into PLA [41,42]. The results revealed that a smaller particle size of 2.7 nm had a 100% bactericidal rate on *L. monocytogenes* and *S. typhymurium* [39]. For particle sizes of 4.5 nm, the bactericidal rates were reported to be 97.04% and 95.85% for *S. aureus* and *E. coli* [39]. However, it must be noted that the difference reported is still small and the tested bacterial strains were different.

Moreover, a study by Scialabba [10] applied a concentration of 1% w/v of AgNPs to chitosan, employing a particle size of 8.05 nm. The bactericidal rate on *S.aureus* and *E.coli* was 20.5% and 23.7%, respectively [10]. Argudín et al. [39] used a particle size of 4.5 nm in PLA which demonstrated a 76.54% increase in antibacterial effectiveness compared to a particle size of 8.05 nm in chitosan [10] for *S. aureus*, as shown in Table 2. However, the difference in bionanocomposites and processing methods differed and these may play a significant role in AgNPs’ antibacterial activity.

The antimicrobial efficiency of AgNP bionanocomposites and in general of bionanocomposites is commonly investigated through different methodologies such as:

- Zone of Inhibition (Agar Diffusion Method): A bioplastic film or disc is placed on an agar seeded with microorganisms and the antimicrobial activity is indicated by the zone of inhibition [37];
- Direct contact test (ASTM E2149 [43]): This test aims to evaluate the antimicrobial activity of the bionanocomposites in direct contact with a suspension of microorganisms. After a selected contact time, the microorganisms are recovered and counted to determine the reduction in viability [44];
- Suspension test (ASTM E2180): Specifically used for hydrophobic bionanocomposites, this method is designed to evaluate the antimicrobial activity of incorporating an active agent in the bionanocomposite, such as ASTM E2149. The bionanocomposite is soaked in a microorganism suspension and the activity is calculated as the percentage of reduced vitality [45].

Microorganisms are the primary culprits behind food spoilage, as they accelerate degradation reactions, alter pH levels, and produce toxins that lead to changes in taste, texture, and appearance, rendering the food unpalatable. Hence, reducing their presence in food products brings several benefits to producers, such as a higher flexibility and time management of food products, as well as to consumers, with better food quality overall and less food spoilage, leading to reduced waste and improving the products’ shelf-life and possible storage periods [46,47].

### 4.2. Enhanced Physical Properties of Bionanocomposite Materials

AgNPs can also improve the physical and functional properties of bioplastic materials, resulting in improved food protection and preservation [41]. The addition of AgNPs into the bioplastic matrix showed improved barrier permeability, mechanical properties, thermophysical stability, and water resistance [41,42]. This is critical since the primary function of food packaging is to extend the food product’s shelf-life by avoiding unfavorable changes caused by microbial breakdown, chemical pollutants, temperature change, air, moisture, light, and external factors [21]. The impact of AgNPs on the physical properties of bioplastic material is critically examined in the following sections.

#### 4.2.1. Membrane Barrier Properties

The barrier properties of packaging contribute to food shelf-life. For example, oxygen scavenging and CO_2_ emission from the pack are moisture-dependent, which is governed in part by the packaging barrier capability. As previously established in the literature, the efficiency of these barrier properties is predominantly contingent upon the primary polymer constituents of the bioplastic materials. The choice of polymers dictates the permeability of the packaging to gases and moisture, directly impacting food’s quality and safety during storage. However, recent advancements in food packaging technology have introduced a transformative factor: the incorporation of AgNPs. AgNPs have garnered significant attention due to their unique properties, which include antimicrobial and barrier-enhancing effects. When AgNPs are integrated into the bioplastic matrix, they interact with the polymer chains, reinforcing the barrier capabilities of the packaging material [47]. AgNPs have shown promising activity in reducing the impact of external factors, particularly the adverse effects of moisture and oxygen on packed food items. This enhancement translates to a tangible reduction in food waste, as the shelf life of products is extended. Additionally, it mitigates potential health risks for consumers, as it minimises the chances of microbial growth and oxidation within the packaging. 

There are several methods to characterise the barrier properties of packaging films, and the most common are: Water Vapour Transmission Rate (WVTR), Water Vapor Permeability (WVP), and Oxygen Transmission Rate (OTR). 

The Water Vapor Transmission Rate (WVTR) is a measurement that quantifies the rate at which water vapour or moisture permeate through a material or barrier over a specific period of time. The WVTR depends highly on the packaging film, thickness, resin composition, and the polymers mixed as well as their physio-chemical properties such as the hydrophilic/hydrophobic degree. This parameter can be improved with the integration of AgNPs by reducing the porosity and increasing the hydrophobicity of the film [48]. Food texture, nutritional, and flavour profiles are all affected by water vapour levels, which have an impact on food quality and safety. When the moisture content of food changes, the rates of lipid oxidation, microbial development, and browning fluctuate [47].

Bahrami noticed a slight but considerable reduction in moisture content with an 8% AgNP concentration, decreasing from 0.2859% to 0.2757% during the study of TG-HPMC-BW films [29]. The increased crosslinking in the biopolymer network caused by the electrostatic contact between the nanoparticles and the OH-groups of glucosyl was responsible for the diffusion of water molecules in the film samples [29]. The moisture content presence was similarly reduced in the agar/banana powder film when AgNPs were integrated, with both blending compositions experiencing a 2% moisture reduction [23].

However, in particular situations, AgNPs may inhibit the intermolecular hydrogen link between polymers. This was observed in the chitosan–starch blend, where the nanoparticles promoted water vapour adsorption at the hydrophilic regions of polysaccharide molecules, and eventually moisture penetration, increasing the WVTR by 24.4% at a 3.79% AgNP concentration [47]. 

AgNPs can influence the WVTR by means of different physical, mechanical, and chemical interactions with both water molecules and the biocomposites. Indeed, besides the described phenomenon, AgNPs can form tortuous paths for water vapour molecules, making it more difficult for them to pass through the biocomposite and thus reducing the WVTR. Moreover, the inherent hydrophobicity of silver core reduces the biocomposite polar behaviour, thus reducing its interaction with water molecules. 

The WVRT of bionanocomposites is commonly quantified through the gravimetric method (ASTM F1249) [49]).

Water Vapor Permeability (WVP) determines how effectively a packaging material can control the moisture transfer between packaged food and its surrounding environment, and its regulation is fundamental to preserve food quality. Its value is measured by means of standard procedures [50]. 

The incorporation of AgNPs positively affects the WVP, resulting in a decrease in the WVP in different compositions. For example, the WVP of the TG-HPMC-BW film fell significantly to approximately half of its original value [51]. This was explained by the presence of AgNPs in the matrix, which prevents the biopolymer chains from moving, resulting in a reduced WVP [12]. Similar behaviour was observed in J. Rhim et al. [20], in which agar, starch, and pectin-based films obtained a 25% reduction with 2% AgNPs. Moreover, Ortega et al. [44] reported a 45% WVP reduction at 28 ppm, and Shankar et al. obtained a 9% reduction at 100 ppm [42]. Although similar results were expected in the agar/banana film, the WVP was increased by 43% through the addition of AgNPs to the sample containing the highest banana powder ratio. This could be due to the reduced compatibility of banana powder and AgNPs, as well as the larger size of the AgNPs detected during SEM imaging [23]. Moura et al. [21] investigated and confirmed this trend while testing different AgNP particle sizes, with 100 nm AgNPs having a 15.8% higher WVP than 41 nm AgNPs in the same cellulose-based composite. Therefore, this shows how smaller-sized AgNPs with larger surface areas enhance the WVP, independently of the concentration of AgNPs employed.

As a result, the addition of AgNPs can improve the composite material’s WVP, with the highest improvement of 53% being observed in the TG-HPMC-BW film, delivering superior results for food preservation through moisture retention [51]. However, the best performing WVP composition of 1.6 × $10^{-10}{g\;m}^{-1}{\;s}^{-1}{\;\mathrm{Pa}}^{-1}$ was a starch-based bionanocomposite at 28.6 ppm AgNPs [52]. 

The Oxygen Transmission Rate (OTR) refers to the measurement of the amount of oxygen that passes through a specific material over a given period. It is a crucial parameter in packaging industries, especially for products that are sensitive to oxygen exposure, such as food. The OTR is typically expressed in cc/m²/day and indicates the material’s barrier properties against oxygen. Measuring the OTR of a film involves oxygen permeation analyzers, which can create a controlled environment. One side of the material is exposed to a high concentration of oxygen, while the other side is exposed to a vacuum or a controlled low oxygen concentration. By monitoring the oxygen permeating through the material, the analyzer calculates the OTR. This measurement is crucial in selecting appropriate packaging materials to ensure the freshness and quality of food products.

Some food shelf-life metrics, such as off-odour, aerobic plate count, and colour parameters, can be affected by the OTR in packaging [53]. It was determined that moderate OTR values (2000–7000 mL O_2_ m^−2^ per 24 h) resulted in the greatest packaging performance. This range of values is commonly seen in synthetic plastics, but bioplastics exhibit a range in the hundreds [54]. The OTR is commonly measured at room temperature at a controlled relative humidity by means of standard procedure [55]. 

Similarly, to the WVP, the OTR of AgNPs containing bionanocomposite demonstrated decreased transmission rates when compared to the original film. As the concentration of AgNPs increased from 0% to 20% in a chitosan–starch-based film, the OTR decreased from 2.39 mL O_2_ m^−2^ per 24 h to 1.48 mL O_2_ m^−2^ per 24 h [19]. Dairi et al. [56] identified this behaviour on a cellulose acetate bionanocomposite, for which the inclusion of 5% AgNP organoclay resulted in a 13.6% drop in the OTR [56]. This behaviour is based on an increase in the diffusion route length, which causes gas molecules to flow more slowly through the matrix [55,56]. 

It can be concluded that the integration of AgNPs into packaging materials offers a versatile means to enhance barrier properties by reducing moisture and oxygen permeation [56]. However, the effects of AgNPs can vary depending on the polymer matrix, characterizing the importance of careful consideration when implementing AgNP-based strategies for food packaging.

#### 4.2.2. Mechanical Properties

The mechanical properties of bionanocomposites, such as tensile strength (TS) and elongation at break (EaB), are generally improved, and are substantially dependent on the AgNP concentration [19,53,54,55]. The improved mechanical capabilities of bionanocomposites can be attributed to their high stiffness and aspect ratio, as well as their strong affinity via contact between the polymer matrix and disseminated nanoparticles. The mechanical properties of nanoparticles in biopolymer food packaging are critical to control, as they allow the food packaging material to resist breakdown and mechanical disruption during manufacturing, transit, and storage [57]. Mechanical characterizations of bionanocomposites offer essential information regarding the composite’s suitability for its intended applications. The characterization procedures adhere to standardized protocols as outlined by ASTM guidelines [58].

Ultimate Tensile Strength (UTS) measures the maximum stress a material can withstand without breaking. In the food packaging industry, UTS is a crucial technique, as it determines the strength and durability of packaging materials, ensuring they can withstand various handling and transportation conditions, thus maintaining the integrity of packaged products.

**Figure 5:**
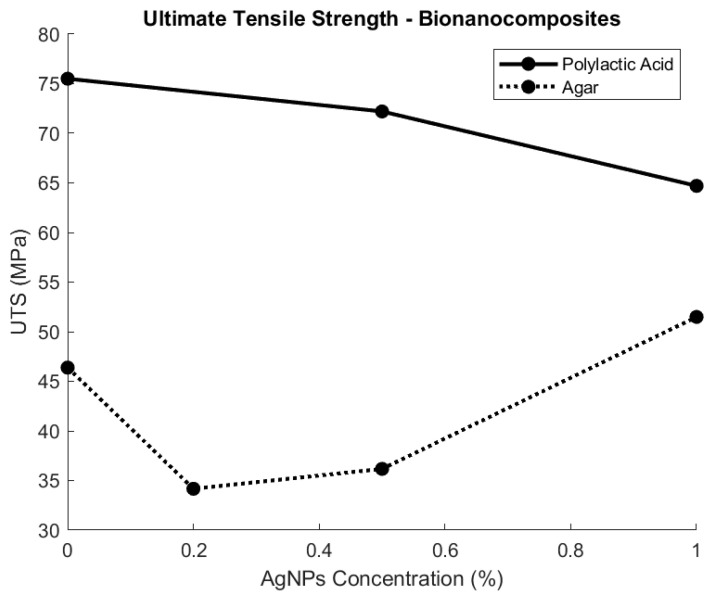
Ultimate Tensile Strength (UTS) of PLA and agar bionanocomposites containing AgNPs [29,52].

Figure 5 summarises two opposite UTS behaviours of the bionanocomposites of PLA and agar polymer matrices, which are dependent on the AgNP concentration. It is important to understand that these two bionanocomposites are used for different packaging applications, as PLA is a hard plastic and the agar-based matrix is flexible plastic. As a hard plastic, PLA will present, by default, a very high UTS, to which the AgNPs’ integration presents a decreasing effect of its structural strength, getting almost 15% lower strength at a 1% AgNP concentration [29]. This is not an isolated case, as it has also been reported that the addition of AgNPs to TG-HPMC-BW reduced the original material’s UTS to nearly half its value [51].

The UTS of the bionanocomposite can be improved through empirical experimentation to determine the optimal AgNP concentration, as observed in agar-based bionanocomposite materials. Although agar-based biocomposites’ initial strength is lower compared to hard plastic materials, the addition of AgNPs (1% concentration) increases the UTS by 8% [20]. This improvement is also seen for chitosan–starch-based films, where the UTS was increased to 69.6 Mpa at a 5.2% AgNP concentration [19]. The UTS of the bionanocomposite material is not only dependent on the base biopolymer and the concentration of AgNPs, but also on the size of the nanoparticles. This was demonstrated by Moura et al., [21] who found that 41 nm-sized nanoparticles increased the UTS of HPMC by 13% while 100 nm particles improved the UTS by only 9.8%.

Therefore, when developing a bionanocomposite, careful consideration of the material design is required when selecting the base material, concentration, and size of the nanoparticles, as this will significantly impact their strength. 

Another key packaging property is the elongation at break (EaB), the material’s ability to stretch before breaking. In food packaging, a high elongation at break is vital as it indicates the flexibility of the packaging material. This property allows packaging to withstand deformations and movements during storage and transportation, preventing tears or ruptures and ensuring the safety and quality of packaged food products.

**Figure 6:**
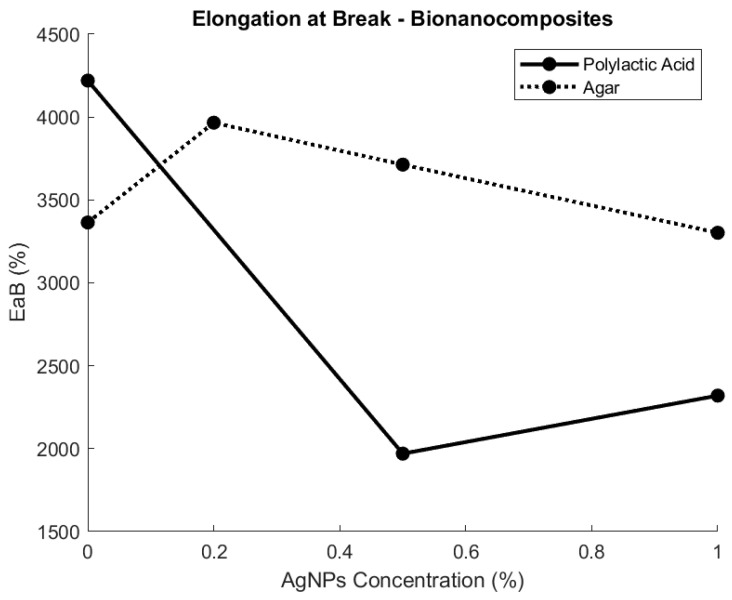
Elongation at Break (EaB) variation in bionanocomposites with AgNP concentration. EaB is a measure of a material’s ability to stretch or deform before breaking, for which hard plastics, such as PLA, typically have a low elongation at break, indicating that they are relatively inflexible and brittle. Flexible plastics such as agar-based composites typically have a higher elongation at break, indicating higher elasticity before breaking [60,61].

The EaB property of the bionanocomposite, similar to UTS, is dependent on the base material, concentration, and size of the nanoparticles. For instance, for materials such as PLA and chitosan/gelatin/polyethylene glycol (CH/GE/PEG), when AgNPs are embedded, the EaB can be reduced by 53%, a significant decrease [30,59]. This enhances the formulation of PLA as a hard plastic by increasing its resistance to external forces, as shown in Figure 6. However, flexible plastic films have the opposite outcome, and the EaB can be increased up to 79% with AgNPs in the case of starch biopolymer films [60,61]. 

In many cases, the addition of AgNPs results in a trade-off between the UTS and EaB [59]. This is visible in HPMC films with different nanoparticle sizes, as both 100 nm and 41 nm nanoparticles decrease the EaB but increase the UTS of the film [51].

Therefore, the specific mechanical requirements will have to be considered when developing the bionanocomposite, and priorities will have to be defined to account for UTS and EaB trade-offs, as illustrated in Table A2. 

#### 4.2.3. Thermal Properties

The thermal properties of bionanocomposites are critical, for example in the industrial thermoforming process for the production of packaging materials. Mixing biopolymers with AgNPs can enhance the thermal and dimensional stability of biopolymers. The enhanced stiffness and reduced thermal expansion that are characteristic of AgNPs have been identified as factors that enhance the dimensional stability of bionanocomposites containing both polymers and nanofillers. Because they have a greater modulus and a lower thermal expansion coefficient than the polymer matrix, bionanocomposites have been reported to have increased dimensional stability [62].

Youssef et al. [13] investigated chitosan embedded with silver and zinc oxide nanoparticles, with silver nanocomposite demonstrating greater thermal stability and reaching a 10% weight loss difference compared to the control composite. Corn starch-based and agar/banana powder bionanocomposites have also shown improved thermal stability, with a 5% and 7% decrease in weight loss, respectively [23,63]. Previous research has shown improvements in PLA thermal stability, respectively, by 8% at 1% wt/wt AgNPs and 6% at 0.3 wt% AgNPs. This improvement was due to the formation of a strong interfacial interaction between the AgNPs and the PLA matrix [64].

As a result, incorporating AgNPs into bioplastic composites has the potential to improve the material’s thermal stability. This is critical for food packaging materials if recycling is a viable option after disposal; high-temperature-resistant materials are more likely to be recycled and reused for packaging, a preferred route to incineration and landfill [65].

#### 4.2.4. Water Resistance Properties

Water resistance is an important characteristic of biodegradable films used in food packaging because, in some situations, the packaging may be exposed to humidity and water during food storage, and the key functions of the packaging may be compromised as a result of the high water activity. Understanding and optimising properties, such as solubility and hydrophobicity, are paramount for ensuring the effectiveness and reliability of biodegradable films in real-world food packaging scenarios.

Food packaging can interact with water in a variety of ways during transit to storage systems such as freezers and fridges. As a result, packaging must be water resistant to a certain extent. Films formed entirely of bioplastics (e.g., chitosan, starch, and sodium alginate) have high solubility rates in water at room temperature, with chitosan and starch having solubility rates of 76% and 21%, respectively [25].

However, in the case of chitosan/gelatin (CH/GE) Ag bionocomposites, the solubility increased by 10% at 0.025% AgNPs concentration, which was explained by the presence of water-soluble phytochemicals such as carbohydrates, alkaloids, and tannins capping AgNPs prepared with Mussaenda Frondosa leaf extract [66]. Alginate films with melanin from watermelon seeds were integrated with zinc oxide and AgNPs to reduce the solubility of the original film. Alginate films had over an 80% solubility, which was decreased to 74% by adding 0.25% melanin. Although zinc oxide nanoparticle integration improved it by 2%, AgNPs had a significant influence, resulting in a nearly 10% drop in water solubility [67]. However, Ortega et al. [44] found that AgNPs significantly augmented the solubility of starch-based films at both 25 °C and 100 °C. Although the bionanocomposite exhibited an 8% lower water solubility at 25 °C, the control film, originally 100% soluble, saw an increase to an average of 45% solubility with just 23 ppm AgNPs at 100 °C [52].

Clear conclusions cannot be drawn due to a lack of consistency in the research and the small volume of research in this area. However, since the increase in solubility with the AgNP concentration was explained by the presence of phytochemicals, the WS might be influenced by the synthesis method and other compounds present in the formulation. Furthermore, the incorporation of AgNPs may result in a more ordered structure in the polymer matrix, preserving the integrity and enhancing the solubility resistance [52].

The Contact Angle (CA) is directly related to the hydrophobicity of the film. A higher CA indicates greater hydrophobicity. When a packaging material is hydrophobic, it repels water-based liquids, preventing moisture from penetrating the package. This is crucial in food packaging, as moisture intrusion can lead to spoilage, mould growth, and reduced food product quality [68]. As a result, a CA target of >90° is set in order to achieve hydrophobic packaging.

Silver nanoparticles have been shown to improve the hydrophobicity of bioplastics in some cases. For example, the CA of agar films was increased by 100% at only a 2% AgNP concentration [23]. This increase in hydrophobicity was also observed in pure chitosan films, which improved the films’ CA by 12% [27]. As a result, the film’s water resistance was improved. 

However, in contrast, blended films of chitosan–starch and agar/banana powder have also shown a lower CA with the addition of AgNPs. This suggests that the biopolymer components within the matrix interacted to a lesser extent, indicating altered surface properties in these composite films. The nanoparticles lowered the CA in the chitosan starch blend film by 15 o at a 20% AgNP concentration [25], and only by 30 in the agar/banana powder mix film [23]. The increased surface roughness due to the presence of AgNPs at the surface could also explain a higher water CA in Ag bionanocomposites. The increased surface roughness creates more microscopic pockets and uneven terrain on the film’s surface, which disrupts water expansion through the film’s surface. 

Considering the above, AgNPs in blended films are likely to increase the hydrophobicity by minimizing the interactions between the biopolymers and by increasing the surface roughness. Plus, the inclusion of an emulsifier component can further enhance the hydrophobicity of individual films. In summary, the water solubility of AgNP bionanocomposites is influenced by various factors, leading to complex outcomes. AgNPs have shown potential in enhancing hydrophobicity, but the effects are influenced by nanoparticle concentration, film composition, and surface roughness changes. These findings emphasise the need for standardised approaches in studying AgNPs’ impact on film properties. Further research is essential to unlock the full potential of AgNPs in efficient and sustainable food packaging materials.

## 5. Bionanocomposites Application as Food Packaging Materials

A variety of foods, including fruits, vegetables, meats, and cheeses, have been tested with bionanocomposites incorporating AgNPs [69]. Silver-based bionanocomposites have been shown to be effective in improving the shelf life of various types of food products, as shown in Table 3.

Highly wasted fruits and vegetables, such as strawberries and carrots, have shown that their shelf life was extended by up to 4 days and 10 days, respectively, compared to untreated samples [72,73]. Another study on the effects of AgNP bionanocomposites on meat, a high-cost and environmentally impactful food product, showed that the storage time could be extended by up to 7 days compared to control [74]. These examples demonstrate the potential of AgNP bionanocomposites to improve the shelf-life of perishable food products and reduce food waste, as premature expiry throughout the food supply chain is a leading cause of waste. Moreover, this application could reduce or eliminate the number of preservatives commonly added directly into the bulk of food, reducing the cost of food and increasing its nutritional quality [75].

## 6. Barriers to Commercial Rollout

### 6.1. Limited Scalability of Bionanocomposites 

The scalability of bionanocomposites depends on various factors such as the manufacturing method, the properties of the virgin material, and the intended application. However, scalability is often a significant challenge for most bionanocomposite production techniques, as summarized in Table 1. 

One scalability barrier is based on the complexity of some of these processes. For example, solution intercalation and in situ polymerization methods often require precise control over the reaction conditions and may involve multiple steps. Changes to the conditions during polymerization can lead to variations in the properties of the bionanocomposite [31,76].

Scalability also depends on the availability and cost of the raw materials, such as the AgNPs and the biopolymer feedstock. For example, producing large quantities of high-quality AgNPs is expensive and time-consuming. Plus, biopolymers are often more expensive than synthetic polymers, since they are typically derived from natural sources and require specific processing and purification steps, which can add to the production costs [77].

Additionally, these processes have challenging optimisation processes. For instance, template synthesis can be sensitive to reaction conditions such as temperature, pH, and solvent choice. The reaction temperature significantly affects the rate of the chemical reactions involved. Higher or lower temperatures can speed up or slow down the process, impacting the final structure’s size, shape, and uniformity. Different reactions have optimal pH ranges where they occur most efficiently. Deviating from this range can lead to incomplete or unwanted reactions, affecting the template synthesis process. Some reactions occur only in specific solvents. The solvent also influences the solubility of reactants and products, affecting the reaction equilibrium and, consequently, the final structure.

In summary, the success of template synthesis relies on maintaining several precise conditions. Any deviation from the optimal conditions might result in undesired structures or incomplete reactions. This can make it difficult to optimise reactions and may limit the range of materials that can be synthesised.

In summary, while some bionanocomposite production methods may be more scalable than others, scalability remains a challenge for most techniques and requires the careful consideration of various factors such as the synthesis method, starting materials, and intended end-use.

### 6.2. Safety & Regulation of Bionanocomposites

As research into the application of nanotechnology in the food sector advances, so does the potential of nanotechnology in the food industry, and hence human exposure to these materials [62]. While multiple studies have indicated that consumers are more ready to accept the use of nanotechnology in food packaging than in food products [62], there is still concern about AgNP migration into food, which poses a risk to consumers’ health above threshold levels [78].

Silver is present in traces of everyday foods, and adults are estimated to eat between 20–80 µg per day [79]. Hence, the migration of silver from the packaging to the food could be of concern as it could further increase this dietary exposure, meaning that the exact silver migration must be quantified [79]. Hence, regulations are in place by governing bodies such as the European Food Safety Authority (EFSA) and the United States food and drug association (USFDA) to regulate the use of AgNPs in active packaging materials [6].

AgNPs should not exceed 0.05 mg/L in water and 0.05 mg/kg in food, according to the EFSA [6]. This means that analysing the migration profile of silver is crucial. It helps ensure the effectiveness of its antibacterial properties and ensures compliance with current regulations [6]. In 2011, the EFSA published a report requiring producers to undergo in vitro genotoxicity, absorption, distribution, metabolism, and excretion testing [6]. Similarly, the USFDA issued a paper with advice for manufacturers of food additives and food contact chemicals [6]. According to the USFDA, companies should conduct research and give a hazard profile for each packaging material containing nanomaterials. Moreover, Canada has no limitations on nanoparticles as additives, with many other countries having little to no food-contact material regulations [7].

The absence of strict regulations on nanoparticles in food-contact materials fosters an environment that is conducive to innovation. This freedom from stringent limitations allows researchers and industries to explore novel applications of nanotechnology in food packaging and other sectors. Innovations in materials science, such as advanced coatings and barrier technologies enabled by nanoparticles, hold the potential to revolutionise food packaging, enhancing the shelf-life, freshness, and overall product quality.

However, this innovation comes with the responsibility of ensuring consumer safety. While the flexibility in regulations promotes creativity and experimentation, it also emphasises the need for robust research on the safety and environmental impact of these emerging technologies. Collaborative efforts between industries, regulatory bodies, and scientific communities can strike a balance between encouraging innovation and safeguarding public health, paving the way for groundbreaking advancements in the field of nanotechnology while ensuring consumer well-being.

Concerning the use of silver nanoparticles in food-contact materials, the EFSA completed a risk assessment in 2021 confirming that the use of AgNPs in polymeric matrices is safe at concentrations of up to 0.025% w/w, corresponding to a total migration of Ag ions that is less than 50 µg/silver per kg of food [61]. As demonstrated by the EFSA study, when 0.025% AgNPs were integrated into polymers employed as food-contact materials, the Ag migration was recorded as 6 µg/kg of food, which is far below the threshold of the 50 µg/kg food-specific migration limit [61].

A study conducted by Echegoyen and Nerín concluded that the migration of Ag from food packaging (testing low-density PE and polypropylene with AgNPs) was increased by 1.43-fold and 50-fold when protecting acidic foods and if the packaging was microwaved, respectively [80]. This indicates that the migration rate is influenced by the type of food packaged and the heating conditions of the packaging. However, this particular study’s results revealed that the migration levels detected were below the threshold set by the EFSA [81]. Moreover, Cushen et al. [82] concluded that the percentage of nanofiller incorporated in the polymer film (testing polyethylene with AgNPs on chicken breast) accelerated Ag migration in comparison to other parameters such as the nanoparticle size, storage time, or temperature conditions. 

Most studies to date have been carried out on synthetic nanocomposites which are by definition highly hydrophobic, enabling low migration levels of AgNPs. However, bioplastics tend to have a higher water absorption capacity, which can make them more prone to AgNP migration [83,84]. Hence, further research on the leaching of AgNPs from bionanocomposites is therefore required.

It is evident that many factors are in play in the migration of AgNP towards food products. Hence, each material containing AgNPs must be evaluated independently with a specific food product stimulant to fully understand the migration risks associated with these materials in order to meet regulatory requirements.

### 6.3. Environmental Considerations of Bionanocomposites 

Around 40% of food packaging materials are made from plastic, with nearly 99% of these plastics being synthetic. Of the remaining 1%, 64% are non-biodegradable while the rest are biodegradable. The use of biodegradable plastics is often seen as a viable alternative to synthetic plastics, but many of these options only break down at high temperatures or when treated in specific industrial composting conditions. While their decomposition is faster than synthetic plastics, their end-of-life environmental impact is still significant due to the production of methane gas from composting, which is 25 times more potent than CO_2_, according to the US Environmental Protection Agency [84].

To evaluate the environmental impact of bioplastics and synthetic plastics, life-cycle assessment (LCA) is a tool used to determine the overall material impact at each stage of their life cycle [85]. This process considers factors such as global warming, human toxicity, abiotic depletion, eutrophication, and acidification, as well as Land-Use Change (LUC)-related emissions, which are important factors to consider when land is converted for composting or biofuel feedstock production [85].

Studies have shown that the use of bioplastics can significantly reduce carbon dioxide emissions compared to petroleum-derived plastics, in the case of PLA by 50–70% [86]. However, the disposal methods currently available, such as incineration or landfilling, are not ideal and the bioplastic’s emissions are significantly dependent on the manufacturing process, for which some are resource-intensive [87]. For example, a study completed by Qinqin Xia indicated that polyhydroxyalkanoates (PHAs) bioplastics could have a reducing effect on global warming potential (−8 × $10^{-5}$ kg CO_2_e per cm^3^/MPa) by almost 2× that of PET LCA, depending on the study’s current manufacturing process [88]. Moreover, in some studies, other categories of the LCA are significantly higher when comparing bioplastics with petroleum plastics. For example, the ozone depletion potential of PLA can be as high as 30 × $10^{-12}$ kg $CFCe^{-11}$ per cm^3^/MPa, while polypropilene (PP) is almost half this value [89]. This difference depends mainly on the selected biopolymer. For example, polybutylene adipate terephthalate (PBAT)’s ozone depletion value is similar to PP, but polybutylene succinate (PBS) has a three-times higher impact. 

**Figure 7:**
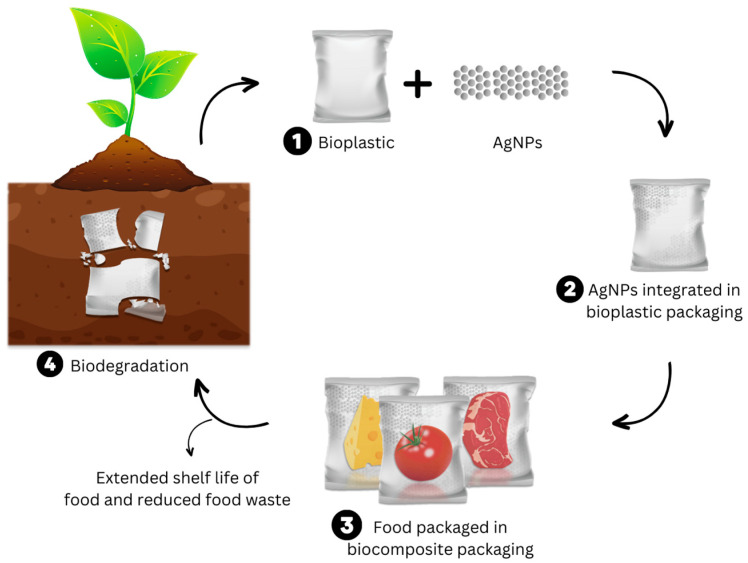
Life cycle of Ag Bionanocomposites. (1–2) Bionanocomposites are materials made by combining natural biopolymers and AgNPs. (3) Bionanocomposites are used as food packaging to extend food shelf-life and improve food safety. (4) Bionanocomposites are then broken down through microbial action in soil, reducing packaging waste compared to synthetic alternatives as well as serving as feedstock for further biomass growth leading towards biomaterial circularity.

Moreover, the integration of nanoparticles in bioplastics adds to their environmental impact, with the upstream production of bulk silver being the dominant factor in nearly every environmental impact category. When AgNPs are applied to bioplastics, the overall environmental burdens are highly sensitive to the synthesis route of the AgNPs. For instance, when applied in wound dressing, the AgNPs could contribute to 66–88% of the global warming impact category depending on the synthesis process [90]. Although the bio-based chemical reduction route was found to have improvements in ozone depletion potential and ecotoxicity, chemical reduction methods are still mainstream and further work into developing green synthesis routes of AgNPs are still required at the commercial scale. A schematic visualization of the life cycle of Ag bionanocomposites is depicted in Figure 7.

Therefore, the replacement of synthetic plastics with bioplastics is challenging, and trade-offs must be considered. The combination of AgNPs and bioplastics with current commercial and large-scale manufacturing presents a significant environmental barrier to a successful and meaningful replacement of synthetic options [91], prompting further research to better understand the full life-cycle impact of these materials.

#### 6.3.1. Bioplastic Fate in the Environment

The accumulation of petrochemical plastic waste in landfills and marine environments poses significant challenges for both living organisms and ecosystems. Additionally, microplastics resulting from the degradation of plastic products have been discovered in unexpected locations, including soil, oceans, seas, drinking water, and even in arctic and mountain regions [92]. This alarming trend, coupled with the escalating levels of CO_2_ emissions in the atmosphere, has compelled the scientific community to explore novel alternatives to traditional plastics, such as biodegradable bioplastics and advanced bionanocomposites.

Bioplastics’ biodegradation can occur under specific conditions depending on their environment and the physical-chemical nature of the bioplastic. In this section, it is important to highlight the difference between the degradation and biodegradation of (bio)plastic materials [93]. Degradation refers to the fragmentation of the polymer chain due to factors like heat, moisture, or enzymes, leading to a loss of polymer structure and the formation of subspecies that may differ from the original monomers [94]. On the other hand, biodegradation involves the complete breakdown of bioplastics into compounds, primarily CO_2_, water, nitrogen (N_2_), or hydrogen (H_2_), carried out by living species such as microorganisms, algae, or more complex organisms [95]. Unlike the residues of conventional plastics, the products of bioplastic degradation should not be toxic and should serve as a source of nutrients for other living organisms [95]. Besides the specific environments where the biodegradation takes place, the bioplastic polymer structure plays a key role during this process. Factors such as surface charge, hydrophilicity, molecular weight, crystallinity, and mechanical and thermal properties significantly influence degradation, particularly when microorganisms are involved [96].

For instance, polylactic acid (PLA), one of the most extensively studied bioplastics, biodegrades under specific conditions of humidity (>60% moisture) and temperature (>50 °C), and in the presence of specific bacteria [97]. The degradation process involves the ester bonds of lactic acid units being hydrolysed. The presence of a semi-crystalline structure in PLA increases its melting temperature, affecting its biodegradability, which predominantly occurs in its amorphous regions [98]. Researchers have explored ways to manage these parameters by combining the PLA with other polymers, such as polysaccharides (e.g., starch, agar, cellulose, and chitosan) or other biopolymers like PHA and collagen [64].

Polymer biodegradation can be tuned and improved by the incorporation of active molecules. In a study conducted by Ramos and colleagues, PLA active bionanocomposite films incorporating a flavor (vanillin) and citrate-stabilised AgNPs (at a concentration of 0.01 mg kg^−1^ of total dry weight) obtained through chemical methods as additives were investigated [99]. A thermal analysis using differential scanning calorimetry (DSC) and thermogravimetric (TG) tests revealed that the introduction of flavour and AgNPs resulted in a decrease in both the glass transition temperature (Tg) and the melting temperature (Tm) of PLA. This decrease in thermal stability demonstrates the potential for improving the degradation process of PLA composite films by altering the polymer’s physical–chemical properties [99]. The inclusion of AgNPs into biocomposites besides the antimicrobial and thermal improvement of polymer matrix has shown promise in successfully accelerating the degradation rate of PLA-based active nanocomposite films, demonstrating promising future uses for the improvement of biocomposites’ biodegradation rate.

#### 6.3.2. AgNPs Fate in the Environment

The degradation of silver bionanocomposites involves the individual degradation processes of each component. After discussing polymer matrix degradation, it is important to focus on AgNPs embedded in the composites. AgNPs do not undergo biodegradation processes, and their fate and environmental transport are influenced by several factors. Their mobility in different environments is linked to water chemistry, including the pH of the suspension and the AgNPs’ physical–chemical features such as size, shape, and the presence of capping agents [100,101]. They may exist as isolated particles in suspension, or they may aggregate, especially in environments with a high ionic strength [102], undergoing partial oxidation, leading to the release of Ag+ ions [103]. Furthermore, AgNPs may react with natural substances present in soils and waters such as sulphide, chloride, and others, altering their original properties [104]. AgNPs can also adsorb charged species in the environment through electrostatic interactions which could lead to their aggregation and the loss of colloidal status [105]. The behaviour of AgNPs is thus significantly influenced by their surface properties and the surrounding environment, including capping agents, methods of synthesis, electrolyte composition, solution ionic strength, pH, and the presence of natural organic matter (NOM) [106]. 

The importance of synthesis methods in AgNPs’ environmental impact is well-supported by life cycle assessment (LCA) investigations, which have demonstrated that physical methods tend to impose higher environmental impacts when compared to chemical and biological synthesis routes, while also reducing costs and energy requirements [107,108].

However, the complexity of this system makes it challenging to definitively anticipate the fate of AgNPs, given the multitude of influencing factors, and in particular, considering the wide number of methods of synthesis and capping agents. Furthermore, the existing literature on this topic does not always provide a consistent picture regarding their environmental impact, fate, and potential toxicity. Researchers continue to seek a definitive answer regarding whether AgNP toxicity arises from the nanoparticles themselves, Ag+ ions, or chemicals involved during their synthesis. In comparison to the well-established field of bioplastic degradation, the study of AgNPs’ fate in the environment is relatively new and still requires further data to better understand the key factors influencing their behaviour. 

#### 6.3.3. AgNPs Environmental Considerations

Notwithstanding the complexity of predicting their fate in the environment, the presence of AgNPs, in particular in soil, appears to offer advantages for seed growth and germination in both laboratory- and field-condition experiments, as they function as growth enhancers, exerting positive effects on various plants [102]. For instance, the use of 20 nm citrate-capped silver nanoparticles obtained through biological methods has been reported to significantly improve bean growth and yield, especially in adverse climatic conditions. Prazak et al. demonstrated a strong beneficial germination effect at various tested concentrations (0.25 and 1.25 mg/mL), coupled with efficient antimicrobial activity [109]. Additional studies have demonstrated the beneficial effects of biosynthesized AgNPs on *Triticum aestivum*, commonly known as wheat. These studies have shown improvements in shoot length, fresh and dry shoot weight, chlorophyll levels, carbohydrate content, and protein content [110]. Similar outcomes have been obtained on different plants such as *Pisum sativum* (common pea) and *Phaseolus vulgaris* (common bean) with AgNPs synthetised through biological methods and stabilised by natural capping agents [111,112]. 

Conversely, other studies have demonstrated the toxicity of AgNPs on various plant species and organisms [102]. In a work by Yin, it has been demonstrated that AgNPs stabilised by Arabic gum synthetized by means of NaBH4 reduction displayed a negative effect when tested at 40 mg/L against Lolium multiflorum. The available data on the toxicity of AgNPs in soil are relatively few and mainly obtained for the AgNP contents of 102 –105 μg/kg soil, which are particularly higher if compared to silver bionanocomposite formulations [113,114]. Numerous studies in the literature consistently highlight a common observation: AgNPs’ synthesis using NaBH4 and/or stabilised by non-natural capping agents consistently exhibits inhibitory effects on various plant species due to the toxicity of the synthetic chemical method [115]. This phenomenon persists across diverse colloidal characteristics, including shape, size, and concentration, resulting in a notable reduction in plant growth [105,116,117,118]. 

The method of synthesis does not only influence the AgNPs’ fate during their degradation but also their possible toxicity against organisms. While chemical and physical methods required toxic reducing and stabilising agents, biological “green” procedures only involve the use of natural and eco-friendly stabilising and reducing agents [77], which confer higher stability and unique biocompatibility key aspects during the bionanocomposites’ biodegradation process [108]. Their enhanced stability avoids particle aggregation and the loss of colloidal behaviour responsible for their possible toxicity in the environment, contributing to AgNPs’ positive effects on plants and lower toxicity [119,120]. Moreover, non-biodegradable stabilizing agents commonly exploited through chemical and physical methods may enhance the AgNPs’ environmental toxicity, supporting the necessity of the development of green methods for metallic nanoparticles [121]. 

#### 6.3.4. Silver Bionanocomposites Degradation

To comprehend the biodegradability of silver bionanocomposites, it is imperative to investigate the individual behaviours of both components, as well as their combined behaviour within the composite material. The biodegradation of silver bionanocomposites is commonly assessed using soil burial tests, similar to those employed for plastic and bioplastic materials, as defined by ISO 20200:2015 [122]. Briefly, the bioplastic is buried in the soil to simulate its deterioration, with the soil’s moisture level maintained by periodic watering. To assess (bio)degradation, samples are periodically taken out (every 15 days) over a 90-day period, and the final dry weight is recorded to determine the weight loss.

The presence of AgNPs, as discussed in this review, can potentially modify the physical–chemical properties of the polymer and influence its degradability due to interactions within the polymeric matrix. When incorporated into alginate films, the introduction of AgNPs leads to a slight decrease in bioplastic biodegradation behaviour, resulting in a 10% reduction in weight loss after 90 days compared to native alginate films [123]. Similar outcomes have been observed with other bioplastics, where the interaction of AgNPs with polysaccharides enhances material properties while slightly reducing the biodegradation rate. In a study conducted by Ediyilyam and co-workers, the reduced biodegradation rate was attributed to the release of AgNPs following biopolymer degradation, which led to the natural reduction in growth of bacterial strains present in the soil [124]. Specifically, their research on chitosan-based bioplastics reinforced with environmentally synthesised 20 nm AgNPs observed a 5% reduction in the biodegradation rate compared to pure chitosan films after a 7-day period. This phenomenon has been observed not only with polysaccharides but also with other biocompatible polymers such as PVA [125], PVA combined with banana peel [126], and various polyesters [127]. 

Despite the inherent challenges associated with this emerging field, the initial findings concerning their biodegradation show significant promise and opportunity. The integration of AgNPs results in only a slight reduction in biodegradability while conferring unique properties that can only be achieved through the use of the noble metal colloids.

## 7. Silver Bionanocomposites Discussion of Open Challenges & Opportunities

When silver nanoparticles are integrated into bioplastics for food packaging applications, these properties, together with a substantial antibacterial action against common foodborne pathogens such as *E. coli*, *S. Aureus*, and *P. aeruginosa*, result in an enhanced food shelf-life. Furthermore, the tunability of bioplastic films by changing the composition by inserting different additives is an appealing concept for food-packaging producers, who may tune the material to guarantee multiple end-uses. Additionally, there is an opportunity to explore novel additives and compositions that can further enhance the performance of these bioplastic films, potentially extending their applications to novel areas beyond food preservations such as cosmetics, medical devices, and pharmaceuticals.

It should be noted that the physicochemical properties and dosage of nanomaterials in foods and food packaging dictate their ultimate fate and safety. The safe use of nanotechnology in food packaging involves rigorous characterization through standard testing, as previously discussed, to quantify the nanoparticle migration and guarantee that it does not exceed the regulatory limits. Moreover, several challenges and considerations accompany the utilisation of silver nanoparticles in food packaging. These challenges encompass scalability issues, environmental impact concerns, cost-effectiveness, and low yields in the production process. Addressing these challenges is essential to ensure the scalability, affordability, and sustainability of incorporating silver bionanocomposites into the food packaging industry. There is an opportunity for research and development efforts to focus on overcoming these challenges and enhancing the viability of silver nanoparticles in food packaging. This could involve innovations in production methods, energy efficiency, and novel cost-effective manufacturing techniques. These multifaceted challenges require dedicated funding for research and development to facilitate the broader and faster adoption of these advanced materials to tackle global challenges such as food waste. Environmental issues are also an important consideration. This includes potential impacts on ecosystems and concerns about the long-term environmental fate of these materials, as reported in Section 6 of this review. Safeguarding the environment in the use of nanotechnology in food packaging involves rigorous characterization through standard testing to assess nanoparticle migration and guarantee that it does not exceed regulatory limits. As the industry advances, there is a growing focus on mitigating the environmental footprint and embracing sustainable processes and practices. One promising avenue is the commercialisation of biological synthesis methods for silver nanoparticles. These approaches offer the potential to reduce the environmental impact associated with traditional production methods. Biological synthesis not only provides a greener and more eco-friendly alternative but also aligns with the broader trend of sustainable and responsible nanotechnology. Further silver technologies which demonstrated good antimicrobial activities and low toxicities are Silver-Based Coordination Polymers (Ag-MOF) (136). In Ag-MOFs, organic ligands encapsulate the metal centre, enabling a controlled, gradual release of metallic species as natural cations, allowing a controlled release of Ag+ ions. However, despite their promising results in terms of stability and antibacterial actions, their integration within bioplastics for food packaging has still not been investigated and could represent a novel alternative to obtaining advanced materials (136).

By actively researching and implementing environmentally friendly approaches through green synthesis and novel technologies, the industry can work towards minimising its ecological footprint and fostering sustainable practices in the development and widespread commercialisation of nanotechnology in food packaging. This not only addresses existing environmental concerns but also positions the industry as a leader in eco-friendly and novel food preservation technologies, which can lead to significant market advantages and consumer trust in products that prioritise environmental responsibility as well as a positive economic impact. Collaborations with environmental organizations can help to verify and communicate the industry’s commitment to eco-friendly practices, fostering a positive public image and promoting industry-wide sustainability initiatives.

## 8. Conclusions and Future Prospects

In this review, it has been reported that the use of silver nanoparticles can improve the antibacterial, mechanical, physical, water-barrier, and thermal properties of bioplastics. As a result, they are becoming increasingly attractive additives to bioplastic formulations in order to generate an ideal food packaging material which has similar properties to synthetic plastics with the added benefits of being biodegradable and non-toxic.

The incorporation of silver nanoparticles increased the ultimate tensile strength by 10–15% across a variety of bioplastics [29,30,31,32], improved the thermal stability through higher melting temperatures [62,63,64], and reduced the Water Vapor Permeability [41,42]. These qualities combine to give the material greater strength and resistance to breaking, and less moisture build-up. The practical application of silver bionanocomposites as food packaging materials has revealed that the shelf-life of meat can be increased by up to 7 days [74]. The shelf-life of food has been demonstrated to increase with the concentration of silver nanoparticles and to differ amongst bioplastic kinds and food products tested. As a result, the ideal concentration of silver nanoparticles can be tuned, providing the necessary increase in food shelf-life while also adhering to safety and regulatory legislation. Moreover, the development of active bioplastics, made from natural resources and grafted with silver nanoparticles, will contribute to reducing the problem of plastic pollution. On the other hand, the addition of silver nanoparticles to the matrix of the film improves multiple bioplastics properties, and, in particular, their antibacterial activity, which makes these active bioplastics a suitable candidate for active food packaging that contributes to less food waste. These bioplastics are a sustainable replacement for highly polluting petroleum-based plastics.

In the future, research should place a strong emphasis on further characterising silver bionanocomposites and gaining a deeper understanding of how these nanoparticles affect key material properties. This research should aim to establish standardised reporting procedures, creating a robust foundation for the development of an entirely new industry centred around these advanced materials.

This forward-looking approach offers food and packaging manufacturers, and, more broadly, fast-moving consumer goods companies (FMCG), a unique opportunity to refine their material properties, with the ultimate objective of achieving optimal product shelf-lives, ultimately fighting the food waste pandemic.

## Figures and Tables

**Table 1:** Summary of bionanocomposites manufacturing processes, outlining main benefits and drawbacks.

Bionanocomposite Manufacturing Method	Benefits	Drawbacks
In situ intercalative polymerization [32]	Improved mechanical, thermal and barrier properties	Only works for low-viscosity polymers.
	Easy to automate	Expensive equipment required.
	Cost-effective materials required	Difficulties in controlling the polymerization, leading to variations in the properties of the bionanocomposite.
Solution intercalation [32]	Homogeneous dispersion.	Environmental concerns around solvent use.
	Increased interlayer spacing of the nanofiller, allowing for greater polymer penetration and adhesion to the filler surface.	Time-consuming processing time.
	Efficient processing.	Nanofiller compatibility issues, could lead to poor dispersion and suboptimal properties of the bionanocomposite.
Template synthesis [32]	Precise control over size and shape.	Template removal is challenging and can damage or alter the properties of the final product.
	Tailored properties.	Sensitivity to reaction conditions lead to low optimised reactions and limited the range of materials.
	High yield.	High cost starting materials.
Melt intercalation [32]	Improved thermal stability of bionanocomposite.	Limited control over intercalation, can result in variations of final product properties.
	Improved barrier properties.	Reduced mechanical strength of end-product.
	Reduced cost compared to other methods.	

**Table 2:** Use of silver nanoparticles in food packaging biomaterials. It has been demonstrated that bionanocomposite materials can help prevent the development of common foodborne pathogens such as *S. aureus*, *E. coli*, *S. typhimurium*, and *L. monocytogenes* at low concentrations.

Polymer Used	Size of AgNPs (nm)	Concentration	Food Tested	Strain Tested	Bactericidal Rate (%)	Ref.
Chitosan	8.05	1% w/v	Data not reported or not investigated	*S. aureus*	20.5%	[5]
				*E. coli*	23.7%	
Polylactic acid	4.5	1% wt/wt	Strawberries	*S. aureus*	97.04%	[32]
				*E. coli*	95.85%	
	2.7	1% wt/wt AgNPs	Fresh apple and apple juice	*L. monocytogenes*	100%	[33]
				*S. typhymurium*	100%	
Alginate	20	0.8%	Strawberries	*S. aureus*	91.26%	[34]
				*E. coli*	92.01%	

**Table 3:** Shelf-life extension of food products packaged in bionanocomposites.

Polymer	Size of AgNPs (nm)	Concentration	Food Tested	Shelf-Life Increase vs. Control	Ref.
Chitosan	80 +/− 11 nm		Meat	1 Week	[69]
		5% w/wcs	Pork	6 days	[70]
			Litchis fruit	2 days	[71]
Polylactic acid	2.5–6.5 nm	1% wt/wt	Strawberries	4 days	[72]
Alginate	5–40 nm	0.25, 0.50, and 1.00 μg/mL	Fior di Latte cheese	5 days	[52]
		50, 60, 70, and 80 μg/mL	Carrot and pear	Up to 10 days	[73]

## Data Availability

Data will be made available on request.

## Nomenclature

AgNP(s)	Silver nanoparticle(s)
CA	Contact angle
CH/GE	Chitosan/Gelatin
CH/GE/PEG	Chitosan/Gelatin/Polyethylene glycol
DSC	Differential scanning calorimetry
EaB	Elongation at break
EFSA	European Food Safety Authority
GT/HMPC/BW	Gum tragacanth/HMPC/Beeswaxes
HPMC	Hydroxypropyl Methylcellulose
LCA	Life cycle assessment
LDPE	Low-density polyethylene
LUC	Land Use Change
MIC	Minimum Inhibitory Concentration
NOM	Natural organic matter
OTR	Oxygen transmission rate
PBAT	Polybutylene adipate terephthalate
PBS	Polybutylene succinate
PHAs	Polyhydroxyalkanoates
PLA	Poly-lactic acid
PP	Polypropilene
TG-HPMC-BW	Tragacanth/Hydroxypropyl methylcellulose/Beeswax
TG	Thermogravimetric
Tg	Glass transition temperature
Tm	melting temperature
TS	Tensile Strength
USFDA	United states food and drug association
UTS	Ultimate tensile strength
WS	Water solubility
WVP	Water vapour permeability
WVTR	Water transmission rate

## Appendix A

**Table A1:** Main bioplastic formulations, with short description, advantages and disadvantages of each component.

Bioplastic	Components	Description	Advantages	Disadvantages	Ref.
TG/HPMC/BW	Gum tragacanth (TG)	Used as emulsifier, thickener, stabiliser and texturant additive.	High water binding ability, efficient suspending action and effective surface-activity	Poor film preparation	[22]
	Hydroxypropyl Methylcellulose (HPMC)	Improve film-forming property	Abundant availability, biodegradability, thermal stability, process ability and excellent film-forming capability	Poor moisture barrier	
	Beeswaxes (BW)	Improve moisture barrier and flexibility	Hydrophobicity and firmly packed crystalline structure		
	Oleic Acid	Emulsifier			
	Glycerol	Plasticizer	Increases flexibility (Elongation at break), low molecular weight non-volatile substance, hydrophilic	Lower moisture retention, decreases UTS, Elasticity modulus, lower oxygen water vapour permeability	
Agar/banana powder	Agar		Good film-forming, abundance, renewability and biocompatability	Low mechanical, water resistance properties	[23]
	Banana Powder	To improve water barrier, UV Screening effect, antioxidant and antimicrobial activity.	Potential to increase hydrophobicity, functional properties to secure food safety and extend its life. Good film-forming. Can help reduce metal ions to nanoparticles.		
	Glycerol	Gelling, stabilisers, emulsifiers, and thickening agents	Temperature stability, firmness directly proportional to agar concentration, good clarity, low adhesiveness and inert. Abundant, cheap. Low hydroscopic property	Low mechanical properties	
Agar	Agar	Gelling, stabilisers, emulsifiers, and thickening agents	Temperature stability, firmness directly proportional to agar concentration, good clarity, low adhesiveness and inert. Abundant, cheap. Low hydroscopic property	Low mechanical properties	[20]
	Glycerol	Plasticizer			
HPMC	HPMC		Good film-forming, good for film or coating. Odorless, water-soluble, and tasteless. Moderate moisture and oxygen permeability.	Low mechanical and barrier properties	[21]
Chitosan–Starch	Starch (Rice and Waxy Rice)	Abundant, non-toxic, renewable source, suitable for film-formation. Cheap, easily biodegradable	Sensitive to Moisture, poor mechanical properties		[25]
	Chitosan	To improve mechanical, water vapor barrier, antimicrobial attributes, reduce cost and biodegradability of starch composite	Abundant, non-toxic, renewable source, suitable for film-formation. Antimicrobial activity		
	Acetic Acid	To dissolve Chitosan flakes			
Chitosan/Gelatin	Chitosan	To improve mechanical properties of Gelatin	Biocompatible, biodegradable, antimicrobial. Better mechanical and gas barrier properties		[82]
	Gelatin	Emulsifier	Gel formation, texturizing, thickening. Good film forming. Absorption of UV light	Low thermal strength, elasticity, mechanical properties	
	Acetic Acid	To dissolve Chitosan flakes			
Chitosan	Chitosan		Antimicrobial activity, edible, excellent oxygen and carbon dioxide barrier	Only acid soluble	[13]
	Formic or Acetic acid	To dissolve Chitosan			
	Poly Ethylene Glycol (PEG)				
	Hydroxyl Ethyl Cellulose		Water soluble, non-ionic, high compatibility		

**Table A2:** Mechanical properties of bionanocomposites.

Bioplastic	Concentration of AgNPs (wt%)	Ultimate Tensile Strength (MPa)	Elongation at Break (%)	Ref.
TG/HPMC/BW	0%	75.5	42.2	[30]
	0.5%	72.2	19.7	
	1%	64.7	23.2	
CH/GE/PEG	0%	24.47	4.48	[82]
	0.0075%	25.80	4.34	
	0.0125%	26.30	4.29	
	0.0250%	26.40	4.12	
Agar	0%	46.38	33.64	[52]
	0.2%	34.17	39.66	
	0.5%	36.17	37.13	
	1%	51.49	33.02	
	2%	53.44	36.46	
Sugar palm starch	0%	0.010	86	[85]
	1%	0.015	165	
	2%	0.020	197	
	3%	0.410	415	
	4%	0.300	376	
